# Bacteraemia caused by non-*faecalis* and non-*faecium Enterococcus* species—a retrospective study of incidence, focus of infection, and prognosis

**DOI:** 10.1007/s10096-023-04690-x

**Published:** 2023-11-03

**Authors:** Roni Lohikoski, Karl Oldberg, Magnus Rasmussen

**Affiliations:** 1 Division of Infection Medicine, Department of Clinical Sciences Lund, Lund University, BMC B14, SE-223 63 Lund, Sweden; 2 Skåne University Hospital, Malmö, Sweden; 3 Department of Clinical Microbiology, Region Skåne, Lund, Sweden; 4 Skåne University Hospital, Lund, Sweden

**Keywords:** Enterococci, Bacteraemia, Infective endocarditis

## Abstract

**Background/aim:**

*Enterococcus faecalis* and *Enterococcus faecium* cause human infections including bacteraemia and infective endocarditis (IE). Only few studies describing non-*faecalis* and non-*faecium Enterococcus* (NFE) infections have been conducted. We aimed to describe the incidence, prognosis, and focus of infection of bacteraemia with NFE.

**Methods:**

This retrospective population-based study included all episodes of patients having a blood culture with growth of NFE between 2012 and 2019 in Region Skåne, Sweden. Information was collected from medical records. Episodes of bacteraemia caused by NFE were compared to episodes of bacteraemia caused by *E. faecalis* and *E. faecium*.

**Results:**

During the study period, 136 episodes with NFE bacteraemia were identified corresponding to an incidence of NFE bacteraemia of 16 cases per 1,000,000 person-years among adults. *Enterococcus casseliflavus* (*n*=45), *Enterococcus gallinarum* (*n*=34), *and Enterococcus avium* (*n*=29) were the most common species. The most common foci of infection were biliary tract infections (*n*=17) followed by gastrointestinal infections (*n*=7). Urinary tract infections were not commonly caused by NFE (*n*=1), and no episodes of IE were caused by NFE. Polymicrobial bacteraemia was more common with NFE (73%) than with *E. faecalis* (35%) and *E. faecium* (42%). Community acquired infections were more common in bacteraemia with NFE compared to *E. faecium*. 30- and 90-day survival rates were 76% and 68%, respectively, and recurrent NFE bacteraemia was seen after 3% of the episodes.

**Conclusion:**

Bacteraemia caused by NFE is rare and is often polymicrobial. Biliary tract focus is common in NFE bacteraemia whereas IE and urinary tract focus are uncommon.

## Introduction

*Enterococcus* is a genus of bacteria known to be commensals of the human gastrointestinal tract and they can occasionally cause severe infections, especially in health care-associated and in nosocomial settings [1–3]. Enterococci can cause a variety of conditions that can be complicated by bacteraemia, such as urinary tract infection, intra-abdominal infections, and infective endocarditis (IE) [4]. The majority of enterococcal infections are caused by the two species *Enterococcus faecalis* and *Enterococcus faecium*, with *E. faecalis* being the most common cause of human infections [1–3]. Other *Enterococcus* spp. known to cause human infections include *Enterococcus casseliflavus*, *Enterococcus gallinarum*, *Enterococcus avium*, *Enterococcus durans*, and *Enterococcus hirae* [5].

About 90% of the cases of enterococcal IE are caused by *E. faecalis*, and *E. faecium* is responsible for most of the remaining cases [6]. The proportion of patients with enterococcal bacteraemia that have IE has been estimated to be between 10 and 30% [7–11].

Bacteraemia caused by different *Enterococcus* species has not been thoroughly investigated, and [12] bacteraemia caused by uncommon enterococci is even less studied. Most of the reports are small case studies such as the one conducted in 2004 by Choi et al. [13] focusing on bacteraemia caused by *E. casseliflavus* and *E. gallinarum*. The first large-scale study with focus on non-*faecalis* and non-*faecium Enterococcus* species (NFE) described 186 episodes of bacteraemia in Taiwan and was presented 2010 by Tan et al. [14]. Tan et al. reported that the most common NFE in bacteraemia were *E. casseliflavus*, *E. gallinarum*, and *E. avium* with the most common types of infection being catheter-associated bloodstream infection, biliary tract infections, and intra-abdominal infections [14].

The aim of this study was to conduct a large retrospective population-based study of all patients with NFE bacteraemia between years 2012-2019 in the Region of Skåne, Sweden, to increase the knowledge on infections caused by these uncommon *Enterococcus* species, to allow for better future work-up and treatment.

## Materials and methods

### Study design and participants

This is a descriptive population-based retrospective study of patients with bacteraemia caused by NFE from 2012 to 2019 in the Region Skåne, Sweden. Information on all episodes with positive blood cultures for *Enterococcus* spp. from January 2012 to December 2019 was obtained from the Department of Clinical Microbiology database of Region Skåne, Sweden. The Department of Clinical Microbiology is the only laboratory in the region which had 1 million inhabitants 18 years and older (December 2012) at the beginning of the study period and 1.08 million (December 2019) at the end [15].

All blood cultures with growth of NFE were included and analysed. From the patients with blood cultures positive for *E. faecalis* and *E. faecium*, a random sample of 100 patients for each species was included for the analysis. The number of episodes in these comparison groups was chosen so that they were more than double the number of episodes in the largest NFE group. The sampling was done by choosing the first episode with a blood culture positive for *E. faecalis* and *E. faecium* every month leading to a total of 96 patients. The remaining 4 patients were randomly chosen from the end of June years 2013, 2015, 2017, and 2019. All the episodes of the patients with *E. faecalis* and *E. faecium* bacteraemia were included in the analysis thus leading to more episodes than total number of included patients.

Patients 18 years of age and over were included in the study. Episodes with incomplete medical history due to transfer to another hospital for treatment were excluded due to lack of information. Multiple positive blood cultures from the same patient were considered as one episode if obtained during the same hospitalisation or 14 days after being hospitalised; otherwise, they were counted as a new episode. One patient can thus contribute with more than one episode.

### Microbiological analysis

A blood culture usually consisted of a set with one aerobic and one anaerobic culture vial. From 2012 to the end of 2014, the blood culture system BacT/ALERT® (bioMérieux, Inc., Durham, NC, USA) was used; from 2015 this system was replaced by BACTEX™ FX (BectonDickinson, Franklin Lakes, NJ, USA). The bacteria were identified using Matrix-Assisted Laser Desorption/Ionisation Time of Flight Mass Spectrometry (MALDI-TOF MS) with the database MBT Compass Library (Bruker Daltonics, Bremen, Germany). A score of 2.0 or higher was required for species identification.

The antimicrobial susceptibility testing for commonly used antibiotics was done by the disc diffusion method according to the European Committee on Antimicrobial Susceptibility Testing (EUCAST), or by determining the minimum inhibitory concentration (MIC) by using gradient strips, Etests (bioMérieux, Marcy l’Etoile, France), on agar plates according to manufacturer’s instructions. The EUCAST clinical breakpoints for *Enterococcus* spp. were used to categorise the isolates as sensitive, intermediate/increased exposure, or resistant according to the valid breakpoint table at the end of the study period [16].

Microbiological parameters included in the analysis were number of blood cultures taken, number of positive blood cultures with enterococci, mono- or polymicrobial bacteraemia, other bacteria isolated, and antimicrobial susceptibility for vancomycin and ampicillin.

### Clinical data

Medical records were studied to obtain relevant patient demographics, medical background, and information on clinical course and outcomes. The collected information consisted of age, sex, comorbidities, number of episodes, microbiological findings, primary focus of infection, site of acquisition, variables included in criteria for IE [17, 18], radiological findings, findings from laboratory, and clinical assessments. At the time of positive blood culture, or within 24 hours from the positive blood culture, the severity of bacteraemia was assessed by calculating the Sequential Organ Failure Assessment (SOFA) score and using the definitions for sepsis as described in Sepsis-3 [19, 20].

To evaluate comorbidities, the Updated Charlson Comorbidity index was calculated [21]. The focus of infection was defined by fulfilment of at least two of the following criteria: (1) typical symptoms of focal infection, (2) isolation of the *Enterococcus* species in question at the site of infection, and (3) radiological signs of a focal infection, as described previously [10]. If less than two criteria were fulfilled, the episode was considered having an unknown focus of infection. Intraabdominal infections were divided into biliary tract infection (cholangitis, cholecystitis, and liver abscess) and gastrointestinal tract infection (gastrointestinal abscess, diverticulitis, appendicitis, ileitis, and post-abdominal surgery infection).

Surgical intervention was defined as any surgical procedure used to gain source control and treat the BSI. Complications to surgery that lead to BSI were not regarded as surgical procedures.

Site of acquisition was defined as nosocomial if a positive blood culture was drawn after 48 hours or more from hospital admission even if this could be due to a doctor’s delay in blood culturing. Health care-associated bacteraemia was defined according to Friedman et al. [22]. Episodes not fulfilling any of these criteria were regarded as community acquired. Modified Duke Criteria were used to assess the episode as definite, possible, or rejected IE [17, 18]. According to the criteria, an episode was regarded to constitute a major microbiology criterion only if there were two or more positive NFE blood cultures in a patient with non-nosocomial bacteraemia with unknown focus.

We also studied if the empiric antibiotic therapy was active against the blood culture isolate. Thus, piperacillin, ampicillin, vancomycin, or benzyl-penicillin or imipenem high doses (3g×3 and 1g×3 respectively) were regarded active against sensitive isolates whereas other empirical treatments were regarded as inactive. Time with antibiotic therapy, treatment at intensive care unit regarding admission and treatment with ventilator, vasopressors and dialysis, the need of surgery, length of stay, and 30- and 90-day survival.

### Statistical data analysis

The data was analysed using IBM SPSS Statistics version 27. The number of inhabitants in the Region of Skåne, Sweden, during the study period was obtained from Statistic Sweden’s website [15]. The incidence was calculated by dividing the number of NFE cases with the mean number of inhabitants from the study period and expressed as number of cases per million inhabitants per year. The Shapiro-Wilk test was performed for all the variables to determine normal distribution, and data was described depending on normal distribution and scale type. Non-normally distributed variables with median and interquartile range (IQR) and categorical variables, for example binary variables, with amount (*n*) and percent (%). To determine statistical significances of differences between groups, chi-squared and 2-tailed Fisher’s exact tests were used for categorical variables and for non-parametric data Mann-Whitney *U* test. The comparison was made only between NFE, *E. faecalis*, and *E. faecium*; NFE were thus not compared with each other nor was *E. faecalis* compared with *E. faecium.* A value of *p* <0.05 was used to define statistical significance.

### Ethics

The Regional Ethics Review Board in Lund, Sweden, granted an ethical approval for the study (2018-898).

## Results

### Epidemiology

Between 2012 and 2019, a total of 2263 episodes of blood cultures with *Enterococcus* species were recorded in the Region of Skåne, Sweden (Table 1). 137 episodes were NFE bacteraemia; one episode was excluded due to transfer to another hospital outside Region Skåne. The most common NFE episodes with bacteraemia were caused by *E. casseliflavus* (*n*=45), *E. gallinarum* (*n*=34), and *E. avium* (*n*=29), followed by *E. hirae* (*n*=10) and *E. durans* (*n*=9). The more uncommon NFE isolates (*E. raffinosus*, *E. thailandicus*, *E. cecorum*, and *E. mundtii*) were analysed as a group “Other *Enterococcus* spp.” (*n*=9). The incidence of NFE bacteraemia was 16 cases per 1,000,000 person-years among adults. Median age for all analysed cases was 74 years, and no significant age differences were found between the different species (Table 2). Bacteraemia with *E. casseliflavus* was significantly more common in females (53%) in comparison to *E. faecalis* (33%) and *E. faecium* (34%).

**Table 1:** Distribution of episodes with *Enterococcus* bacteraemia in Region Skåne, Sweden, 2012–2019

*Enterococcus* species	Episodes (*n*)	Proportion (%)
*Enterococcus faecalis*	1402	62
*Enterococcus faecium*	725	32
*Enterococcus casseliflavus*	45	2.0
*Enterococcus gallinarum*	34	1.5
*Enterococcus avium*	29	1.3
*Enterococcus hirae*	10	0.4
*Enterococcus durans*	9	0.4
*Enterococcus raffinosus*	4	0.2
*Enterococcus thailandicus*	3	0.1
*Enterococcus cecorum*	1	0.04
*Enterococcus mundtii*	1	0.04
Total:	2263	100

**Table 2:** Patient demographics, microbiology, severity of infection, and site of acquisition

	All NFE (*n*=136)	*Enterococcus casseliflavus* (*n*=45)	*Enterococcus gallinarum* (*n*=34)	*Enterococcus avium* (*n*=29)	*Enterococcus hirae* (*n=*10)	*Enterococcus durans* (*n*=9)	Other *Enterococcus* spp.^**1**^ (*n*=9)	*Enterococcus faecalis* (*n*=132)	*Enterococcus faecium* (*n*=119)
Demographics									
Age, median (IQR)^*^	75 (60–84)	78 (62–85)	73 (65–84)	70 (59–77)	78 (57–87)	68 (60–75)	71 (67–86)	75 (66–81)	71 (64–79)
Female sex, *n* (%)	**68 (50)**^**A,B**^	**24 (53)**^**A,B**^	17 (50)	14 (48)	6 (60)	2 (22)	5 (56)	44 (33)	41 (34)
uCCI, median (IQR)^*^	2 (0–6)	2 (1–6)	3 (0–6)	3 (1–6)	2 (0–2)	2 (0–3)	2 (2–6)	2 (1–3)	2 (1–6)
Microbiology									
≥2 pos blood cultures, *n* (%)	**73 (54)**^**A**^	24 (53)	22 (67)	12 (41)	7 (70)	4 (44)	4 (44)	96 (73)	64 (54)
Polymicrobial infection, *n* (%)	**99 (73)**^**A,B**^	**37 (82)**^**A,B**^	**22 (65)**^**A,B**^	**23 (79)**^**A,B**^	6 (60)	6 (67)	5 (56)	46 (35)	50 (42)
Severity of infection									
SOFA Score, median (IQR)^*^	3 (2–4)	**3 (2–4)**^**A**^	3 (1–4)	2 (1–4)	2 (0–3)	3 (2–7)	3 (2–4)	2 (1–3)	2 (1–4)
Sepsis (SOFA≥2), *n* (%)	**103 (76)**^**B**^	39 (87)	24 (71)	19 (66)	5 (50)	8 (89)	8 (89)	89 (67)	72 (61)
Site of acquisition^2^									
Community acquired, *n* (%)	**49 (36)**^**A,B**^	**19 (42)**^**A,B**^	**10 (29)**^**B**^	**10 (35)**^**B**^	4 (40)	3 (33)	3 (33)	29 (22)	8 (7)
Health care-ass, *n* (%)	**53 (39)**^**A**^	19 (42)	14 (41)	12 (41)	3 (30)	2 (22)	3 (33)	79 (60)	46 (39)
Nosocomial, *n* (%)	**34 (25)**^**B**^	7 (16)	10 (29)	7 (24)	3 (30)	4 (44)	3 (33)	24 (18)	65 (55)

*NFE* non-*faecalis* and non-*faecium Enterococcus* species, *IQR* interquartile range, *uCCI* updated Charlson Comorbidity Index, *SOFA* Sequential Organ Failure Assessment

Values in bold represent those that are different from *E. faecalis* (A) and *E. faecium* (B) with statistical significance (*p* < 0.05, defined in methods)

*Non-normally distributed variable

^1^Other *Enterococcus* spp. include *E. raffinosus* (4), *E. thailandicus* (3), *E. cecorum* (1), and *E. mundtii* (1)

^2^The comparison was not performed between *E. hirae*, *E. durans*, and other *Enterococcus* spp. and *E. faecalis* or *E. faecium* due to the limited number of cases

^A^Significant difference compared to *E. faecalis*

^B^Significant difference compared to *E. faecium*

### Microbiology and site of acquisition

Polymicrobial infections were significantly more common in bacteraemia with *E. casseliflavus*, *E. gallinarum*, and *E. avium* in comparison to both *E. faecalis* and *E. faecium* (Table 2)*.* In bacteremia with all *Enterococcus* species, the most common site of acquisition was health care-associated (46%), but in bacteraemia caused by NFE community acquired infections were more common than in bacteraemia caused by *E. faecalis* and *E. faecium* (Table 2).

### Focus of infection and infective endocarditis

Unknown focus was more common than known focal infection in NFE bacteraemia (69% versus 31%) (Table 3). The most common known foci of infection were biliary tract infection and gastrointestinal tract infection. *E. avium* was found to cause significantly more gastrointestinal infections in comparison to *E. faecalis* and *E. faecium*. *E. casseliflavus*, *E. avium*, and *E. durans* were significantly more common agents for biliary tract infections in comparison to *E. faecalis*. A significantly lower proportion of urinary tract infections were found in bacteraemia with *E. casseliflavus*, *E. gallinarum*, and *E. avium* in comparison to *E. faecalis* (Table 3).

**Table 3:** Focus of infection

	All NFE (*n*=136)	*Enterococcus casseliflavus* (*n*=45)	*Enterococcus gallinarum* (*n*=34)	*Enterococcus avium* (*n*=29)	*Enterococcus hirae* (*n*=10)	*Enterococcus durans* (*n*=9)	Other *Enterococcus* spp.^**1**^ (*n*=9)	*Enterococcus faecalis* (*n*=132)	*Enterococcus faecium* (*n*=119)
Unknown focus, *n* (%)	**108 (79)**^**A**^	**40 (89)**^**A,B**^	**28 (82)**^**A**^	19 (66)	7 (70)	6 (67)	8 (89)	73 (55)	85 (71)
Gastrointestinal infection^**2**^, *n* (%)	7 (5)	0 (0)	0 (0)	**5 (17)**^**A,B**^	1 (10)	1 (11)	0 (0)	1 (0.8)	6 (5)
Biliary tract infection^**3**^, *n* (%)	**17 (13)**^**A**^	**5 (11)**^**A**^	4 (12)	**4 (14)**^**A**^	2 (20)	**2 (22)**^**A**^	0 (0)	4 (3)	7 (6)
Urinary tract infection, *n* (%)	**1 (1)**^**A,B**^	**0 (0)**^**A,B**^	**1 (3)**^**A**^	**0 (0)**^**A**^	0 (0)	0 (0)	0 (0)	39 (30)	11 (9)
Other focus, *n* (%)^**4,5**^	**3 (2)**^**A,B**^	0 (0)^**A**^	1 (3)	1 (3)	0 (0)	0 (0)	1 (11)	15 (11)	10 (8)
Infective endocarditis, *n* (%)	**0 (0)**^**A**^	0 (0)	0 (0)	0 (0)	0 (0)	0 (0)	0 (0)	8 (6)^**6,7**^	1 (1)^**7**^

*NFE* non-*faecalis* and non-*faecium Enterococcus* species

Values in bold represent those that are different from *E. faecalis* (A) and *E. faecium* (B) with statistical significance (*p* < 0.05, defined in methods)

^1^Other *Enterococcus* spp. include *E. raffinosus* (4), *E. thailandicus* (3), *E. cecorum* (1), and *E. mundtii* (1)

^2^Gastrointestinal infection includes abdominal abscess, diverticulitis, post-surgical abscess, appendicitis, and ileitis

^3^Biliary tract infection includes liver abscess, cholangitis, and cholecystitis

^4^Other focus includes respiratory tract infection, wound, skin, soft tissue, bone or joint, and catheter infections

^5^Two patients that had more than one focus of infection were counted as other focus. The first had urinary tract infection and other foci. The second had urinary tract infection and spondylodiscitis

^6^Using the new Duke-ISCVID [23] criteria, two additional episodes of IE with *E. faecalis* were identified

^7^Transesophageal echocardiography was performed in 26 *E. faecalis* and 10 *E. faecium* episodes*.* Transthoracic echocardiography was performed in 50 *E. faecalis* and 40 *E. faecium* episodes

^A^Significant difference compared to *E. faecalis*

^B^Significant difference compared to *E. faecium*

Transthoracic echocardiography was performed in 112 patients, of whom 22 patients had NFE bacteraemia (16% of all NFE episodes), and transoesophageal echocardiography was performed in 41 patients of which 5 patients had NFE bacteraemia (4% of all NFE episodes). No episodes with IE caused by NFE were found (Table 3). IE was found in eight episodes of *E. faecalis* bacteraemia (6% of all episodes with *E. faecalis*) and in one episode of *E. faecium* bacteraemia (1%) (Table 3).

### Antibiotic susceptibility

Most enterococci were sensitive to ampicillin apart from *E. faecium* where only 13% were sensitive (Table 4). One *E. avium* isolate and one *E. faecium* isolate were resistant to vancomycin (Table 4). Empiric therapy with effect against the isolate was given in 63% of episodes with NFE bacteraemia (Table 5).

**Table 4:** Antibiotic susceptibility

	All NFE (*n*=136)	*Enterococcus casseliflavus* (*n*=45)	*Enterococcus gallinarum* (*n*=34)	*Enterococcus avium* (*n*=29)	*Enterococcus hirae* (*n*=10)	*Enterococcus durans* (*n*=9)	Other *Enterococcus* spp.^**1**^ (*n*=9)	*Enterococcus faecalis* (*n*=132)	*Enterococcus faecium* (*n*=119)
Ampicillin									
Sensitive, *n* (% of tested)	**129 (96)**^**A,B**^	**45 (100)**^**B**^	**34 (100)**^**B**^	**28 (97)**^**B**^	**8 (89)**^**B**^	**9 (100)**^**B**^	**5 (56)**^**A,B**^	132 (100)	15 (13)
Resistant, *n* (% of tested)	6 (4)	0 (0)	0 (0)	1 (3)	1 (11)	0 (0)	4 (44)	0 (0)	104 (87)
Not tested, *n*	1	0	0	0	1	0	0	0	0
Vancomycin									
Sensitive, *n* (% of tested)	**52 (41)**^**A,B**^	**0 (0)**^**A,B,2**^	**0 (0)**^**A,B**^	26 (96)	9 (100)	8 (100)	9 (100)	127 (100)	118 (99)
Resistant, *n* (% of tested)	74 (59)	39 (100)	34 (100)	1 (4)	0 (0)	0 (0)	0 (0)	0 (0)	1 (1)
Not tested, *n* (%)	10	6	0	2	1	1	0	5	0

*NFE* non-*faecalis* and non-*faecium Enterococcus* species, *MIC* minimum inhibitory concentration

Values in bold represent those that are different from *E. faecalis* (A) and *E. faecium* (B) with statistical significance (*p* < 0.05, defined in methods)

^1^Other *Enterococcus* spp. include *E. raffinosus* (4), *E. thailandicus* (3), *E. cecorum* (1), and *E. mundtii* (1)

^2^According to EUCAST *E. casseliflavus* and *E. gallinarum* should always be reported as resistant to vancomycin without testing [16]

^A^Significant difference compared to *E. faecalis*

^B^Significant difference compared to *E. faecium*

**Table 5:** Prognosis including antibiotic and other treatment, 30- and 90-day survival, and recurrence

	All NFE (*n*=136)	*Enterococcus casseliflavus* (*n*=45)	*Enterococcus gallinarum* (*n*=34)	*Enterococcus avium* (*n*=29)	*Enterococcus hirae* (*n*=10)	*Enterococcus durans* (*n*=9)	Other *Enterococcus* spp.^**1**^ (*n*=9)	*Enterococcus faecalis* (*n*=132)	*Enterococcus faecium* (*n*=119)
Empiric therapy with effect against the isolate, *n* (%)	**85 (63)**^**B**^	**29 (64)**^**B**^	18 (53)	**19 (66)**^**B**^	4 (40)	**9 (100)**^**A,B**^	6 (67)	75 (57)	45 (38)
Total time with antibiotics in days, median (IQR)^*^	**13 (9–18)**^**A,B**^	**13 (9–16)**^**B**^	**12 (10–16)**^**A,B**^	**14 (10–19)**^**B**^	15 (13–20)	19 (9–26)	13 (9–20)	15 (11–21)	19 (12–34)
Days of IV antibiotics, median (IQR)^*^	**9 (6–18)**^**B**^	8 (5–11)	10 (6–14)	8 (5–16)	9 (5–12)	10 (6–19)	9 (8–10)	7 (5–13)	15 (9–29)
Days of P.O. antibiotics, median (IQR)^*^	**4 (0–9)**^**A**^	4 (0–10)	0 (0–7)	5 (0–9)	8 (5–13)	0 (0–6)	0 (0–7)	7 (0–10)	4 (0–10)
ICU-admission, *n* (%)	19 (14)	6 (13)	3 (9)	5 (17)	1 (10)	**4 (44)**^**A,B**^	0 (0)	14 (11)	17 (14)
Surgical interventions, *n* (%)	58 (43)	23 (51)	9 (27)	14 (48)	3 (30)	5 (56)	4 (44)	45 (34)	50 (42)
Total length of stay, median (IQR)^*^	**14 (8–21)**^**B**^	**11 (7–17)**^**B**^	**16 (12–22)**^**B**^	**18 (8–28)**^**B**^	**13 (10–20)**^**B**^	19 (8–54)	11 (10–26)	12 (7–26)	30 (14–53)
30-day survival, *n* (%)	**104 (76)**^**A**^	35 (78)	**24 (71)**^**A**^	22 (76)	8 (80)	6 (67)	9 (100)	115 (87)	91 (76)
90-day survival, *n* (%)	92 (68)	31 (69)	21 (62)	19 (66)	7 (70)	6 (67)	8 (89)	102 (77)	75 (63)
Recurrence within 6 months, *n* (%)	**4 (3)**^**A,B**^	**1 (2)**^**A,B**^	**1 (3)**^**A**^	**0 (0)**^**A,B**^	1 (10)	0 (0)	1 (11)	24 (18)	17 (14)

*NFE* non-*faecalis* and non-*faecium Enterococcus* species, *IQR* interquartile range, *ICU* intensive care unit

Values in bold represent those that are different from *E. faecalis* (A) and *E. faecium* (B) with statistical significance (*p* < 0.05, defined in methods)

*Non-normally distributed variable

^1^Other *Enterococcus* spp. include *E. raffinosus* (4), *E. thailandicus* (3), *E. cecorum* (1), and *E. mundtii* (1)

^2^The comparison was not performed between *E. hirae*, *E. durans*, and other *Enterococcus* spp. and *E. faecalis* or *E. faecium* due to the limited number of cases

^A^Significant difference compared to *E. faecalis*

^B^Significant difference compared to *E. faecium*

### Treatment, prognosis, and outcome

The median length of stay in hospital was 14 days for all the episodes with NFE, and episodes caused by *E. casseliflavus*, *E. gallinarum*, *E. avium*, and *E. hirae* had significantly shorter length of stay in comparison to *E. faecium* (Table 5). Fourteen percent of episodes with NFE required admittance to ICU (Table 5). Surgical intervention was needed in 43% of episodes (Table 5). The most common procedures were endoscopic retrograde cholangiopancreatography, nephrostomies, and different types of surgical drainages. The median time with antibiotic treatment was 13 days (Table 5). The 30-day survival rate was 76%, and the 90-day survival rate was 68% (Table 5). Recurrence within 6 months from concluded antibiotic treatment occurred in 3% of episodes (Table 5). *E. casseliflavus*, *E. gallinarum*, and *E. avium* had significantly lower recurrence rates in comparison to *E. faecalis* (Table 5).

## Discussion

This study demonstrates that NFE bacteraemia occurs with an incidence of 16 cases per 1,000,000 person-years in adults in our region and that *E. casseliflavus*, *E. gallinarum*, and *E. avium* are the most frequently encountered species. *E. casseliflavus* and *E. gallinarum* were also reported by Tan et al. to be the most common species in NFE [14]. The most common enterococcal blood isolates in our study were *E. faecalis* (62%) and *E. faecium* (32%) accounting for 94% of all episodes of enterococcal bacteraemia, and this is also in line with previous studies [24, 25]. The proportion of episodes of enterococcal bacteraemia caused by NFE was lower in our study (6%) than reported previously from Taiwan by Tan et al. [14] and from South-Korea by Choi et al. [13], but higher than reported from the USA by Patterson et al. [24] and from Canada by Billington et al. [25].

Bacteraemia with enterococci is often either health care-associated or nosocomial [12, 24, 26, 27]. We report that a relatively high proportion, 36%, of all episodes with NFE bacteraemia were community acquired which is similar to the proportion in previous reports [13, 14, 25, 28].

Previously reported common foci of infection for NFE are the biliary tract, intravascular catheters, and gastrointestinal tract [13, 14, 28]. It is worth noticing that urinary tract infections are rare with NFE in comparison to *E. faecalis* and *E. faecium.* The results of this study follow a similar pattern of biliary tract and gastrointestinal infections being the most common foci, while at the same time most of the episodes with NFE bacteraemia have unknown focus. This might be due to our strict criteria for a focal infection even though Tan et al. [14] used similar criteria based on clinical, microbiological, and radiological findings. A higher proportion of polymicrobial infections can also indicate that the primary focus of infection is intra-abdominal in many episodes of bacteraemia caused by NFE. It is worth noticing that, in this study, clinicians often identified a possible focus of infection based on their clinical evaluation and started therapy without performing detailed radiological investigations.

We did not find any episodes of NFE-caused IE. IE has been reported to occur with NFE bacteraemia in some studies [14, 28, 29]. Since echocardiography was only performed in a minority of patients with NFE bacteraemia we cannot exclude the possibility that cases of IE might have been missed. However, since relapses were uncommon, we find it unlikely that many cases of IE were missed. From our results and earlier studies, it can be concluded that IE does not commonly occur in NFE bacteraemia, and we find it reasonable to suggest echocardiography to be performed only in cases of NFE bacteraemia where a specific suspicion of IE is raised. Regarding IE caused by bacteraemia with *E. faecalis* we found a lower proportion (6%) than reported before [8–12, 29, 30]. IE caused by *E. faecium* was uncommon, as found also in previous work by Anderson et al., Lwin and Bannan, and by Pinholt et al. [9, 11, 12]. The recently updated Duke-ISCVID criteria do not include non-*faecalis Enterococcus* species as a typical cause of IE [23], and our results are compatible with the classification of *E. faecalis* as a typical IE-pathogen and the non-*faecalis* enterococci as non-typical pathogens.

Regarding ampicillin resistance, our findings are comparable to Choi et al. [13] for *E. gallinarum* but in our study all *E. casseliflavus* isolates were sensitive. Our data show that empiric therapy with effect against the isolate was applied only in around half of the episodes, which is similar as reported before by Souhail et al. [31], but much higher than that reported by Pinholt et al. [12].

We report a 30-day survival of 76% which is higher than most previous reports [14, 26]. Factors such as comorbidities, different routines, and antibiotic susceptibility might influence the risk of death but also the likelihood of drawing blood cultures from patients presenting with relatively mild symptoms of infection will affect the proportion of survivors in different studies.

Strengths of this study include that it is large and one of the most comprehensive studies focusing on bacteraemia caused by NFE. The population-based approach gives a low risk for selection bias. Weaknesses include the retrospective design which is a limitation leading to lack of some relevant clinical data and microbiological information. Another limitation is that only a random sample of *E. faecalis* and *E. faecium* was included in the analysis.

As the dataset was large and multiple statistical comparisons were performed, there is also a risk for a multiple comparison problem. We tried to lower this risk by only making comparisons between NFE and *E. faecium* and *E. faecalis* avoiding comparisons between episodes caused by different NFE species and between episodes caused by *E. faecalis* and *E. faecium*.

In conclusion, we show that NFE bacteraemia is rare, more often community acquired, and can be distinguished from bacteraemia with *E. faecalis* and *E. faecium* by a higher proportion of polymicrobial infections and a higher proportion of infectious foci in the biliary tract. Urinary tract infections leading to bacteraemia and IE with NFE are very rare.

## Data Availability

Anonymized data will be shared upon reasonable request.
